# Supplementary material for: Enhanced flexoelectricity at reduced dimensions revealed by mechanically tunable quantum tunnelling
Source: Nat Commun. 2019 Feb 1;10:537. doi: 10.1038/s41467-019-08462-0 (PMC6358620; doi:10.1038/s41467-019-08462-0)
Supplement: Supplementary file 1 — Supplementary Information [file 41467_2019_8462_MOESM1_ESM.pdf]

Enhanced flexoelectricity at reduced dimensions revealed by mechanically tunable quantum tunnelling

Saikat Das et al.

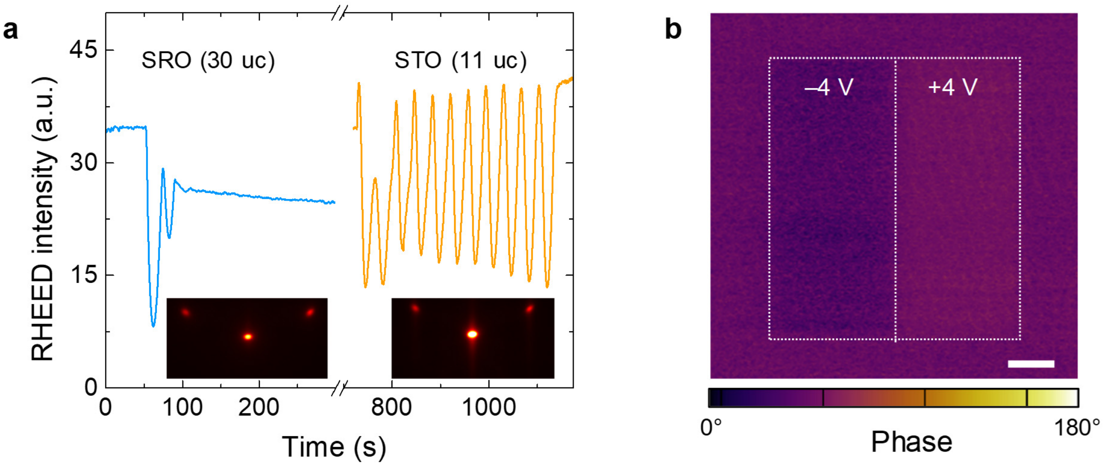

Supplementary Figure 1. Growth and electrical characterisation. **a,** In situ growth monitoring employing reflection high-energy diffraction (RHEED). The growth mode of the metallic $SrRuO_3$ (SRO) layer exhibited the typical transition from layer-by-layer to step-flow, as evident by the evolution of RHEED intensity (blue curve). The $SrTiO_3$ (STO) layer, however, grew in a layer-by-layer manner. Here, we show representative RHEED intensity profiles collected during the growth of 30 unit cell-thick SRO and 11 unit cell-thick STO layers. The RHEED patterns obtained after growth (shown underneath the intensity profiles) feature sharp Bragg reflexes, indicative of atomically smooth surfaces. **b,** The piezoresponse force microscopy (PFM) phase image of an eleven unit cell-thick STO film taken after electrical poling with ±4V of applied bias. No phase contrast is discernible across the poled area, suggesting that our STO film was paraelectric. We obtained similar PFM phase images for the nine- and five-unit cell-thick films. Source data are provided as a Source Data file.

Supplementary Note 1

To extract barrier heights from the tunnelling *I*–*V* curves, we used an analytical equation describing direct tunnelling through trapezoidal tunnel barriers[1,2]:

$$I(V) \cong b + c\frac{\exp\left\{\alpha(V)\left[(\varphi_2 - \frac{eV}{2})^{\frac{3}{2}} - (\varphi_1 + \frac{eV}{2})^{\frac{3}{2}}\right]\right\}}{\alpha^2(V)\left[(\varphi_2 - \frac{eV}{2})^{\frac{1}{2}} - (\varphi_1 + \frac{eV}{2})^{\frac{1}{2}}\right]^2}\sinh\left\{\frac{3}{2}\alpha(V)\left[(\varphi_2 - \frac{eV}{2})^{\frac{1}{2}} - (\varphi_1 + \frac{eV}{2})^{\frac{1}{2}}\right]\frac{eV}{2}\right\}, \quad (1)$$

where c is a constant and $\alpha(V) \equiv [4d(2m_e)^{1/2}]/[3\hbar(\varphi_1 + eV - \varphi_2)]$. Also, b, m_e, d, and $\varphi_{1,2}$ are the baseline, the electron mass, barrier width, and barrier height, respectively.

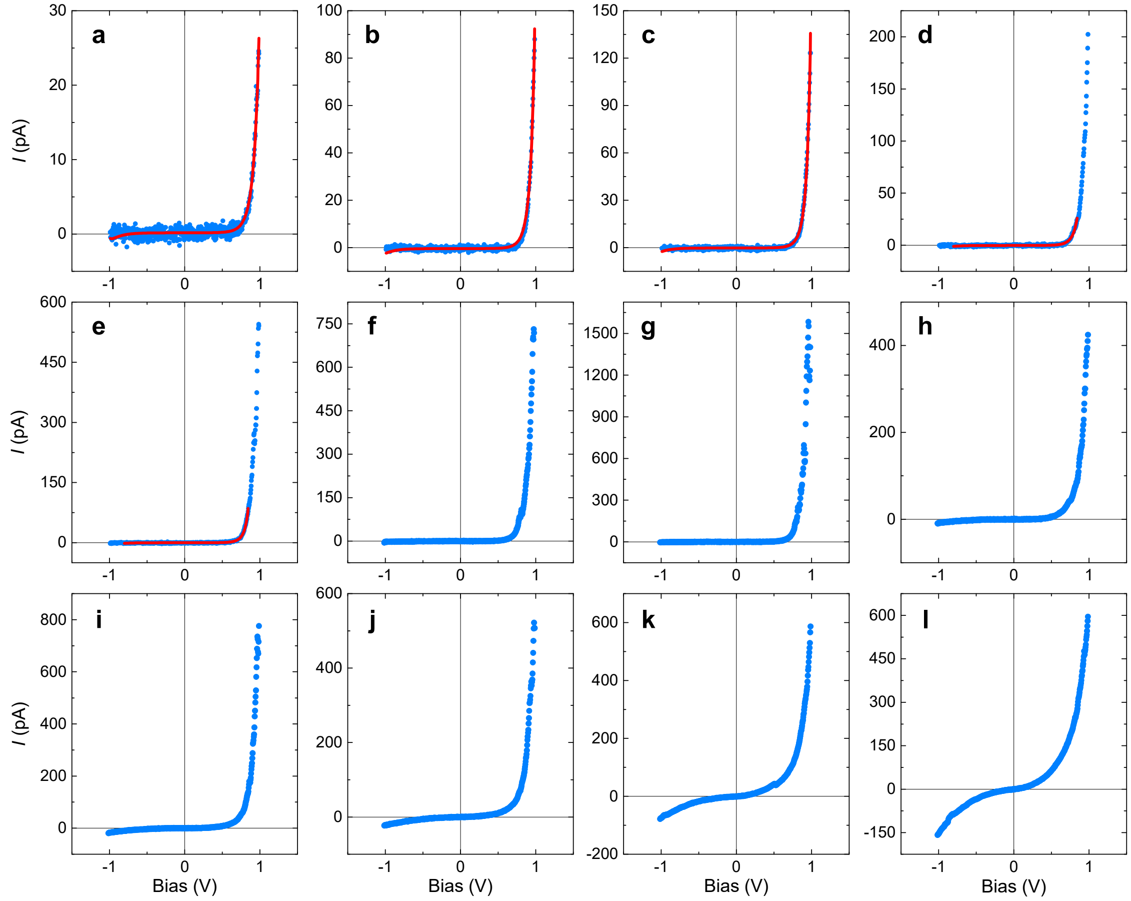

Supplementary Figure 2. Tunnelling currents across a nine-unit cell-thick $SrTiO_3$ film with increasing strain gradients. Tunnelling currents were measured as the applied strain gradient increased from 1.14×10^7 m^{-1} (**a**) to 1.7×10^7 m^{-1} (**l**). Figure 2 of the main text presents spectra **a**, **f**, and **l**. The solid red lines in Figures **a**–**e** indicate the fits to Supplementary Eq. 1. Note that in **a**–**c**, we fitted the entire spectra (i.e., –1 V to +1 V), but we used smaller bias windows to fit the tunnelling currents of **d** and **f**. Source data are provided as a Source Data file.

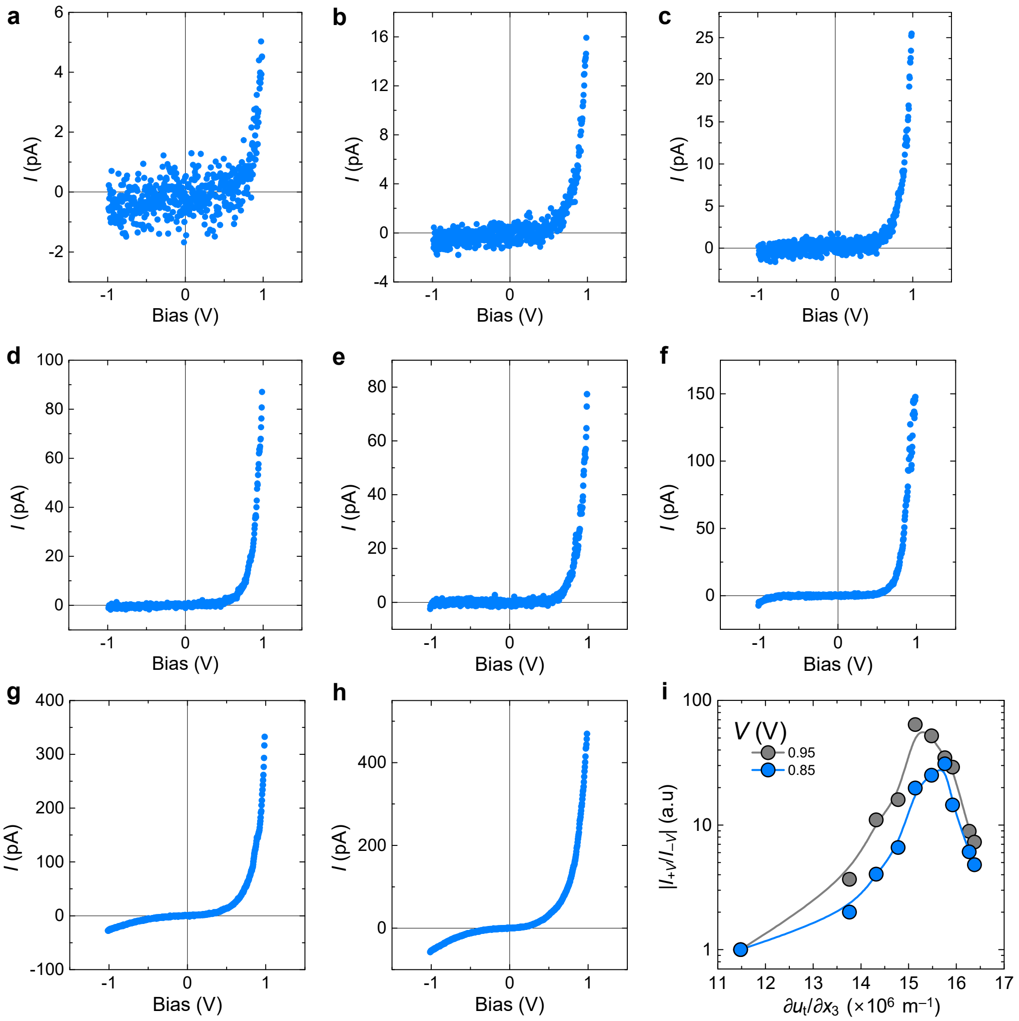

Supplementary Figure 3. Tunnelling currents across an eleven-unit cell-thick $SrTiO_3$ film. **a**–**h**, Tunnelling currents measured as the applied strain gradient increased from 1.15×10^7 m^{-1} (**a**) to 1.63×10^7 m^{-1} (**h**). **i**, *RRs*, $|I_{+V}/I_{-V}|$, plotted as a function of $\partial\varepsilon_t/\partial x_3$, showing the asymmetric-symmetric crossover. We used two biases, 0.95 and 0.85 V, for calculation. Source data are provided as a Source Data file.

Supplementary Note 2

We considered an STO thin film of thickness h_f, with the top surface in contact with an AFM tip and the bottom interface coherently constrained by the substrate. At the top surface, the normal stress distribution (as a function of the distance from the contact center) is described by the Hertz contact mechanics of the spherical indenter, as follows:

$$\sigma_{33}^{\text{tip}}(r)=\begin{cases}-\dfrac{3p}{2\pi a^2}\sqrt{1-\dfrac{r^2}{a^2}}, & r\le a\\ 0, & r\ge a\end{cases}, \tag{2}$$

where $a=\left(\frac{3}{4}\frac{pR}{E^*}\right)^{\frac{1}{3}}$ is the contact radius determined by a loading force p, a tip radius R, and an effective Young's modulus E^*. The latter describes the stiffness of the tip-film contact pairing via $1/E^* = (1 - \upsilon_{\text{film}}^2)/E_{\text{film}} + (1 - \upsilon_{\text{tip}}^2)/E_{\text{tip}}$, where E and υ are Young's modulus and the Poisson ratio, respectively. At the film-substrate interface, the displacement is continuous for coherency and is assumed to relax to zero within a depth of h_s into the substrate (i.e., $\eta_i|_{x_3=-h_s}=0$). The clamping effect of the STO substrate is considered to render the average strain zero at each layer of the film (i.e., $\overline{u_{11}}=\overline{u_{22}}=0$ and $\overline{u_{12}}=0$). Finally, the boundary value problem of elastic equilibrium, assuming no body force, is given by:

$$\begin{cases}\sigma_{ij,j}=0\\ \sigma_{33}|_{x_3=h_f}=\sigma_{33}^{\text{tip}},\ \sigma_{31}|_{x_3=h_f}=\sigma_{32}|_{x_3=h_f}=0\\ \eta_i|_{x_3=-h_s}=0\end{cases}, \tag{3}$$

where stress is related to strain via $\sigma_{ij}=c_{ijkl}e_{kl}=c_{ijkl}(u_{kl}-u_{kl}^0)$. The eigenstrain u_{ij}^0 is derived from strain-order parameter couplings of STO through $u_{ij}^0=Q_{ijkl}P_kP_l+\lambda_{ijkl}q_kq_l$, where Q_{ijkl} and λ_{ijkl} are the electrostrictive and rotostrictive tensors, respectively. Using the Khachaturyan microelasticity theory and the Stroh formalism of anisotropic elasticity, we obtained the displacement field of the entire system and then the strain and stress distributions.

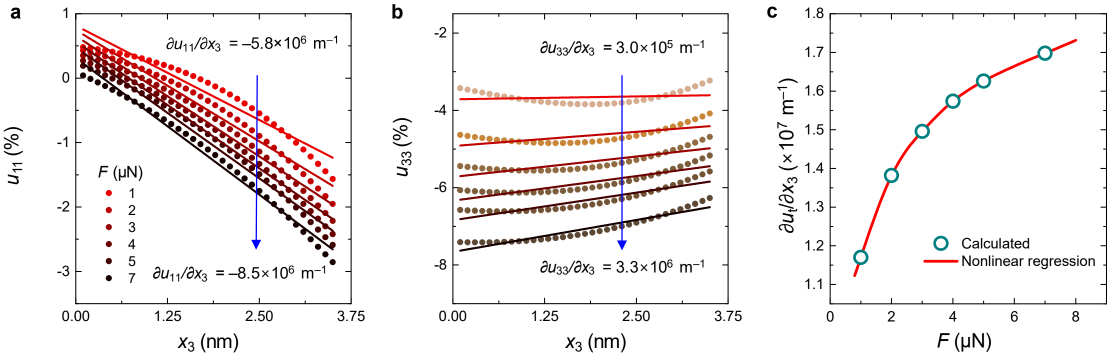

Supplementary Figure 4. Calculated strain gradients imposed by the AFM tip. a, The transverse strains (u_{11} values) under the tip (solid circles). The solid lines are the linear fits to the data. The slopes correspond to the transverse strain gradients ($\partial u_{11}/\partial x_3$). **b,** The longitudinal strains (u_{33} values) under the tip (solid circles). The solid lines are the linear fits to the data**,** and the slopes are the longitudinal strain gradients ($\partial u_{33}/\partial x_3$). Calculations were performed for various applied forces in the range 1–7 μN. **c,** The calculated total transverse strain gradients, $\partial u_t/\partial x_3 = \partial u_{11}/\partial x_3 + \partial u_{22}/\partial x_3$ (open circles). The solid red line indicates the interpolated/extrapolated strain gradients. Source data are provided as a Source Data file.

Rigorously, the Hertz contact mechanics assumes a non-frictional contact between two isotropic, elastic materials. For dielectric materials, such as the incipient ferroelectric STO, it is generally not linear elastic because of the presence of electromechanical couplings (piezoelectric, flexoelectric, and electrostrictive effects) as well as antiferrodistortive-strain couplings (rotostrictive effect). However, we use the Hertz contact mechanics only to obtain the stress distribution at the STO film surface. With this surface stress distribution as the top boundary condition (and zero displacements at the substrate bottom as the bottom boundary condition), we calculated the stress distribution in the whole system (the film and the substrate) by solving the mechanical equilibrium equation (Supplementary Eq. 3). In our simulation, we also self-consistently take into account the electrostrictive coupling (thereby piezoelectric effects), flexoelectric coupling, and rotostrictive coupling as eigenstrains (stress-free strains). This approach allows us to extend reliably the Hertz contact mechanics to the flexoelectric materials for obtaining stress/strain distribution under the force imparted by the tip.

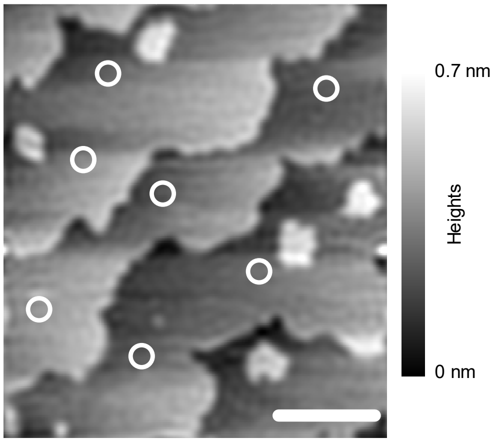

Supplementary Figure 5. Topography image after tunnelling measurements. Open circles mark the locations where we performed the force dependent tunnelling measurements. This image elaborates that the applied force and bias do not cause either mechanical deformation or electrochemical formation. The scale bar represents 500 nm.

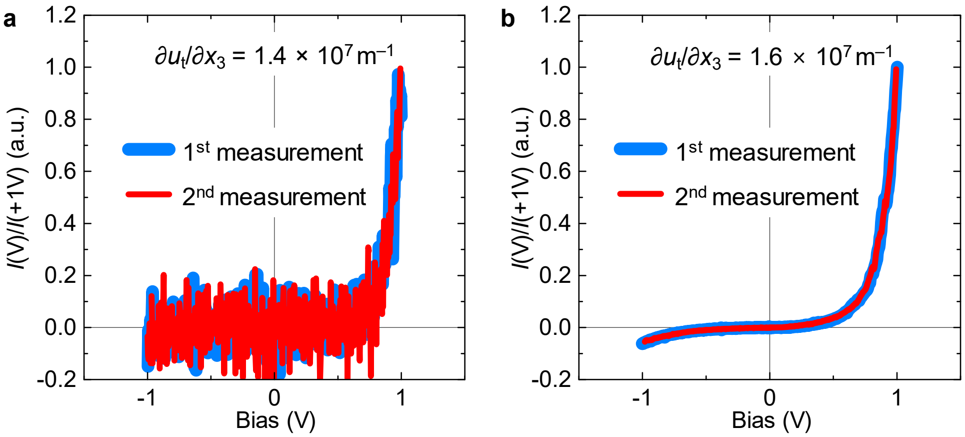

Supplementary Figure 6. Reversible mechanical control of electron tunnelling. Normalized tunnelling currents measured across a nine-unit cell-thick $SrTiO_3$ (STO) film with $\partial u_t/\partial x_3$ of 1.4 × 10^7 m^{-1} (**a**) and 1.6 × 10^7 m^{-1} (**b**). Blue and red lines indicate the data collected during 1st and 2nd measurements at the same site. Source data are provided as a Source Data file.

Supplementary Note 3

We investigated the effect of strain imposed by the AFM tip. Based on our analytical modeling, the surface stress induced by the AFM tip increased compressive strain by a few % in both the longitudinal and transverse directions. This increase could modify two physical features of the STO layer: (1) the band gap and (2) the physical thickness. First, the band gap of STO increased slightly under compressive strain (decreasing the crystal volume)[3]. Also, according to our strain analysis (Supplementary Fig. 4), pressing by the AFM tip decreased the physical thickness of STO by a few %. We thus incorporated strain-induced systematic changes into the tunnel barrier profiles (Supplementary Figs. 7a,b). However, even after these changes, the $|I_{+1\text{ V}}/I_{-1\text{ V}}|$ values increased only negligibly (Supplementary Fig. 7c). Therefore, any effect of strain per se does not explain our experimental observations.

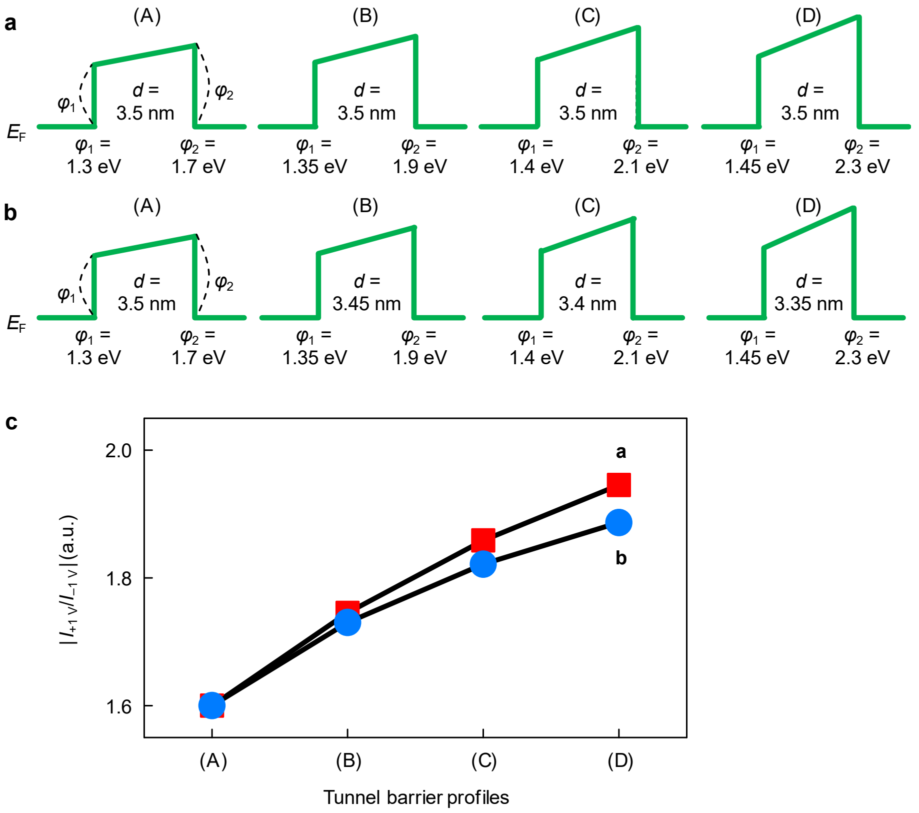

Supplementary Figure 7. The negligible effect of strain per se. a, **b**, Systematic modifications of the trapezoidal tunnel barriers. The strains are larger near the STO/PtIr interface than the SRO/STO interface. Thus, any strain-induced increase in the $SrTiO_3$ (STO) band gap would be larger near the STO/PtIr interface, leading to a greater increase in φ_2 than φ_1. In **b**, we consider the strain-induced systematic decrease in the barrier width *d*. **c**, *RRs*, (i.e., $|I_{+1\text{ V}}/I_{-1\text{ V}}|$ values) of the tunnel barrier profiles in **a** and **b**, calculated using Supplementary Eq. 1. $\varphi_{1,2}$ represent the barrier heights.

Supplementary Note 4

Using the one-dimensional WKB approximation, we can simply describe the tunnelling current density for a low T and small V, as follows:

$$\begin{aligned} j(V) &= \frac{2e}{h}\int_{-\infty}^{\infty} T(E)\times[f(E)-f(E-eV)]dE \\ &\cong \frac{2e}{h}\int_{-\infty}^{\infty} \exp(-\frac{4\pi}{h}\int_0^d \sqrt{2m(U(x)-E)}dx)\times[f(E)-f(E-eV)]dE\,, \\ &\cong \frac{2e}{h}\exp(-\frac{4\pi}{h}\int_0^d \sqrt{2m(U(x)-E_F)}dx)\times eV \end{aligned} \quad (4)$$

where $T(E)$, $f(E)$, and $U(x)$ represent the transmission probability, the Fermi-Dirac distribution, and the tunnel barrier profile, respectively.

Using Supplementary Eq. 4, we obtain the tunnelling current density for a trapezoidal barrier profile (Supplementary Fig. 8), as follows:

$$\begin{aligned} j(+V) &= \frac{2e}{h}\exp(-\frac{4\pi}{h}\int_0^d \sqrt{2m\left\{\frac{\varphi_2-\varphi_1+eV}{d}(x-d)+\varphi_2\right\}}dx)\times eV \\ &= \frac{2e}{h}\exp(-\frac{8\pi\sqrt{2m}}{3h}\cdot d\cdot\frac{(\varphi_2)^{1.5}-(\varphi_1-eV)^{1.5}}{\varphi_2-\varphi_1+eV})\times eV \end{aligned}\,, \quad (5)$$

$$\begin{aligned} j(-V) &= -\frac{2e}{h}\exp(-\frac{4\pi}{h}\int_0^d \sqrt{2m\left\{\frac{\varphi_2-\varphi_1-eV}{d}(x-d)+\varphi_2-eV\right\}}dx)\times eV \\ &= -\frac{2e}{h}\exp(-\frac{8\pi\sqrt{2m}}{3h}\cdot d\cdot\frac{(\varphi_2-eV)^{1.5}-(\varphi_1)^{1.5}}{\varphi_2-\varphi_1-eV})\times eV \end{aligned}\,. \quad (6)$$

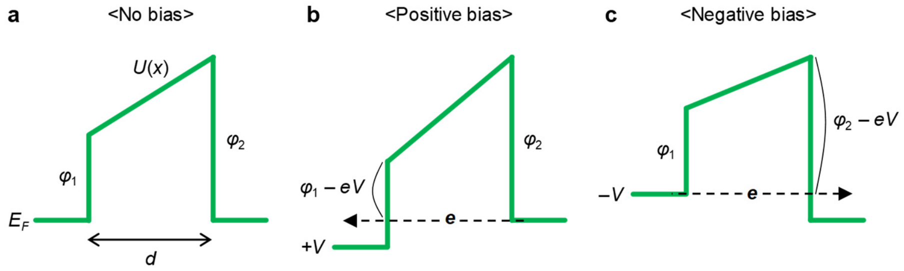

Supplementary Figure 8. Trapezoidal tunnel barriers. Schematics of a trapezoidal tunnel barrier under no bias (**a**) and under positive (**b**) and negative (**c**) bias. $\varphi_{1,2}$ represent the barrier heights.

Using Supplementary Eq. 4, we obtain the tunnelling current density for a triangular barrier profile (Supplementary Fig. 9), as follows:

$$\begin{aligned} j(+V) &= \frac{2e}{h}\exp\left(-\frac{4\pi}{h}\int_0^{d'}\sqrt{2m\left\{\frac{\varphi}{d'}(x-d')+\varphi\right\}}dx\right)\times eV \\ &= \frac{2e}{h}\exp\left(-\frac{8\pi\sqrt{2m}}{3h}\cdot d\cdot\frac{\varphi^{1.5}}{\varphi+eV}\right)\times eV \end{aligned}, \qquad (7)$$

$$\begin{aligned} j(-V) &= -\frac{2e}{h}\exp\left(-\frac{4\pi}{h}\int_0^{d}\sqrt{2m\left\{\frac{\varphi-eV}{d}(x-d)+\varphi-eV\right\}}dx\right)\times eV \\ &= -\frac{2e}{h}\exp\left(-\frac{8\pi\sqrt{2m}}{3h}\cdot d\cdot(\varphi-eV)^{0.5}\right)\times eV \end{aligned}. \qquad (8)$$

Finally, using Supplementary Eqs. (5–8), we obtain tunnelling *I–V* curves for systematically modified tunnel barrier profiles (Supplementary Fig. 10). In these calculations, we assume d = 3.5 nm.

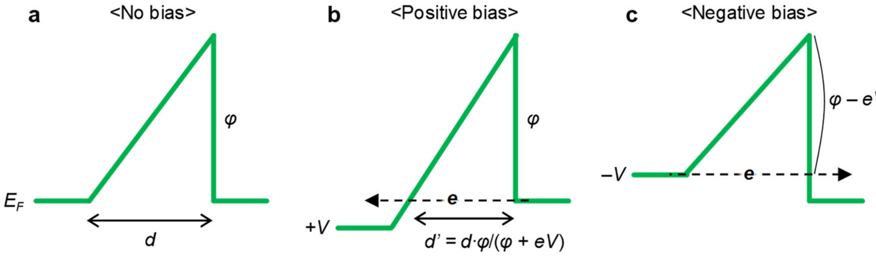

Supplementary Figure 9. Triangular tunnel barriers. Schematics of a triangular tunnel barrier under no bias (**a**) and under positive (**b**) and negative (**c**) bias. φ represents the barrier height.

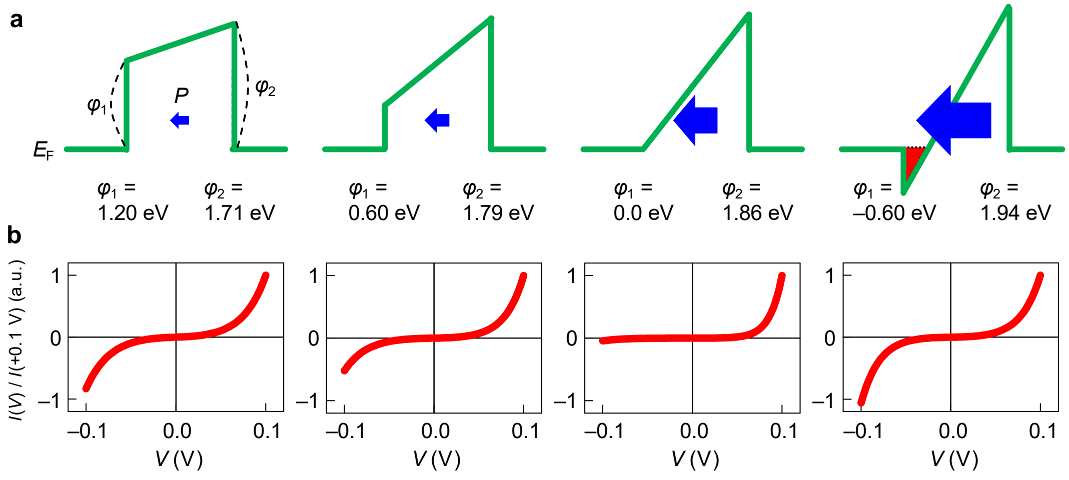

Supplementary Figure 10. Systematic changes in tunnel currents. Tunnel barrier profiles (**a**) and the corresponding tunnelling *I*–*V* curves (**b**), calculated using the Wentzel–Kramers–Brillouin (WKB) approximation. $\varphi_{1,2}$ represent the barrier heights.

Supplementary Note 5

To understand the effect of the electronic polarisation to the interfacial electronic structure, we constructed SRO/STO bilayer with 5 unit cells of SRO and 9 layer of STO, part of which is shown in Fig. 3a. The sub-interfacial layers of completely relaxed paraelectric phase of STO on SRO are insulating as can be seen from the plot of layer resolved density of states (LDOS) by black continuous lines as shown in Fig. 3b, in which Fermi level lies in the gap between the conduction band minima and valance band maxima. However, when STO is polarised, the induced field bends bands and brings the bottom of conduction bands of sub-interfacial STO layers below Fermi level, as shown by blue filled curves in the plot of LDOS (Fig. 3b). We plot Fig. 3 with frozen uniform displacement of Ti atom by 0.2 Å. Note that polarised tetragonal STO have higher energy than paraelectric cubic STO, but can be stabilized in non-equilibrium strain conditions[4]. This band profile clearly supports the experimental finding that the metallized interfacial STO layer changes the barrier profile from trapezoidal to triangular.

Supplementary Note 6

To understand how the strain affects the band structure of $SrRuO_3$ (SRO) and subsequently the tunnelling transport, we additionally performed first-principles DFT calculations. We fixed the in-plane lattice parameter of SRO to that of STO substrate, and imposed compressive strain u_{33} (ranging from 0 to –8%) in the out-of-plane direction. This assumption closely accounts for the strain distribution, obtained from the phase-field simulations (Supplementary Fig. 4). As shown in the Supplementary Figs. 11a and b, our calculation suggests that with increasing the strain, the density of states at the Fermi energy (ρ_F) slightly increases and thus the screening length ($\delta_{SRO} \propto 1/\sqrt{\rho_F}$) could decrease, whereas the work function of SRO (W_{SRO}) slightly decreases by ~0.2 eV. Given the electrostatic constraint $\Delta\varphi_1/\Delta\varphi_2 = (\varphi_{0,1} - \varphi_1)/(\varphi_2 - \varphi_{0,2}) = \delta_{SRO}/\delta_{PtIr}$, where $\varphi_{0,1}$ is proportional to W_{SRO}, the influence of the decreased δ_{SRO} on the tunnel barriers seems to cancel out that of the decreased W_{SRO}. Furthermore, these changes in δ_{SRO} and W_{SRO} are too small to be responsible for the anomalous behavior of tunnel transports (Supplementary Fig. 11c). Thus, we conclude that the effect of strain on SRO is not significant.

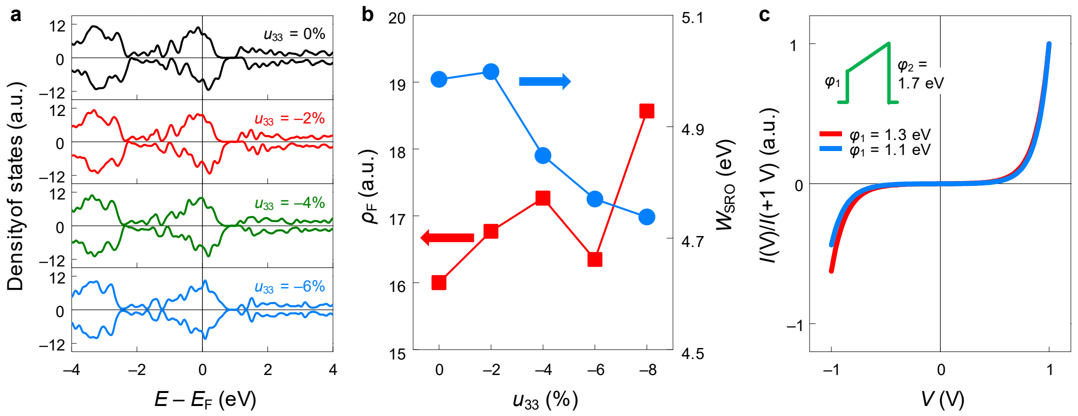

Supplementary Figure 11. Effect of strain on the $SrRuO_3$ electrode. a, Density of states of $SrRuO_3$ (SRO) for different out-of-plane strains u_{33}. **b**, Density of states at the Fermi energy (ρ_F; red squares) and work function (W_{SRO}; blue circles) of SRO as a function of u_{33}. **c**, Tunnelling I–V curves, corresponding to the tunnel barrier profiles (inset), calculated using Supplementary Eq. 1.

Supplementary Note 7

For an ultrathin polarised STO layer sandwiched by SRO and PtIr metals, free carriers in SRO and PtIr partly screen the surface charges in the polarised STO layer. Considering the screening charge, the flexoelectric polarisation should modify the tunnel barrier profile according to the following electrostatic equations[5,6]:

$$(\varphi_2 - \varphi_1)/ed = (P - \sigma_s)/\varepsilon + E_{bi} = [f_{eff} \cdot (\partial u_t/\partial x_3) - \sigma_s/\varepsilon] + E_{bi}, \quad (9)$$

$$\sigma_s = d \cdot P/[(\varepsilon/\varepsilon_0) \cdot (\delta_{SRO} + \delta_{PtIr}) + d], \quad (10)$$

where σ_s is the magnitude of the screening charge per unit area. Given $\varepsilon/\varepsilon_0 = 40$, $\delta_{SRO} + \delta_{PtIr} = 0.7$ nm, and $d = 3.5$ nm, Supplementary Eq. 10 estimates σ_s to be as small as $0.11P$. For an order-of-magnitude estimate of f_{eff} in the main text, therefore, we just used the simplified electrostatic equation 1 in the main text, where we neglect the effect of σ_s. If we additionally consider the effect of σ_s, the linear slope (i.e., 23 ± 1 V) in the $(\varphi_2 - \varphi_1)/ed$ vs. $\partial u_t/\partial x_3$ curve (Fig. 4b) corresponds to $(1 - 0.11)f_{eff} = 0.89f_{eff}$. Thus, this correction gives $f_{eff} = (23/0.89)$ V = 26 V.

Supplementary Note 8

At the critical polarisation, P_c, we expect that $\varphi_1 = 0$ and $\varphi_2 = \varphi_{0,2} + \varphi_{0,1}\cdot(\delta_{PtIr}/\delta_{SRO}) =$ 1.7 + 1.3/8 eV, yielding $(\varphi_2 - \varphi_1)_c/ed = 5.32 \times 10^8$ V m^{-1}. With $E_{bi} = 9 \times 10^7$ V m^{-1}, obtained from fitting (Fig. 3b), we can roughly estimate the critical polarisation as $P_c = \varepsilon\cdot[(\varphi_2 - \varphi_1)_c/ed - E_{bi}] \sim 0.156$ C m^{-2}, where we used $\varepsilon = 40\varepsilon_0$ based on the average strain state (i.e., $u_{33} = -0.06$ and $u_{11} = u_{22} = -0.01$; Supplementary Fig. 4) and DFT calculation (Supplementary Fig. 12).

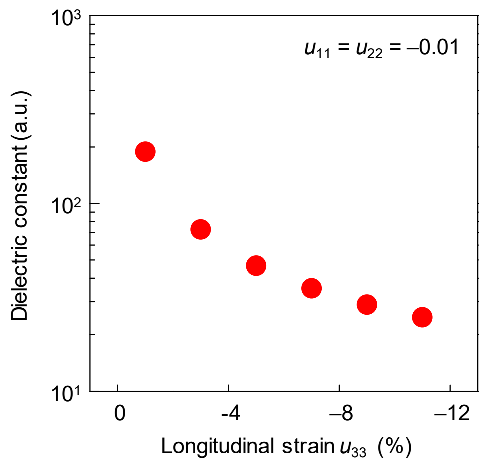

Supplementary Figure 12. Calculated dielectric constant as a function of longitudinal strain. Density functional theory (DFT) calculations of the out-of-plane component of the total dielectric constant (i.e., ε_{zz}), which includes both ionic and electronic contributions, as a function of strain u. The strain was measured with respect to the DFT equilibrium lattice of 3.86 Å.

Supplementary Note 9

A nonlinear flexoelectric response could arise under large strain gradients, as demonstrated in several material systems[7,8]. In the case of a centrosymmetric material like STO, the quadratic flexoelectric term should be zero, so we additionally considered the cubic flexoelectric term, i.e., $P/\varepsilon = f\cdot(\partial u_t/\partial x_3) + g\cdot(\partial u_t/\partial x_3)^3$, where f and g are the first-order and third-order flexocoupling coefficients. For simplicity, by assuming f = 2.6 V (i.e., bulk flexocoupling coefficient)[9], we fitted our data and found that g is minuscule, as small as 3.8×10^{-14} V m^2 (Supplementary Fig. 13). However, when $\partial u_t/\partial x_3$ is huge, e.g., much larger than 10^5 m^{-1}, the effective flexocoupling coefficient, i.e., $f_{eff} = (P/\varepsilon)/(\partial u_t/\partial x_3) = f + g\cdot(\partial u_t/\partial x_3)^2$, might become significantly enhanced.

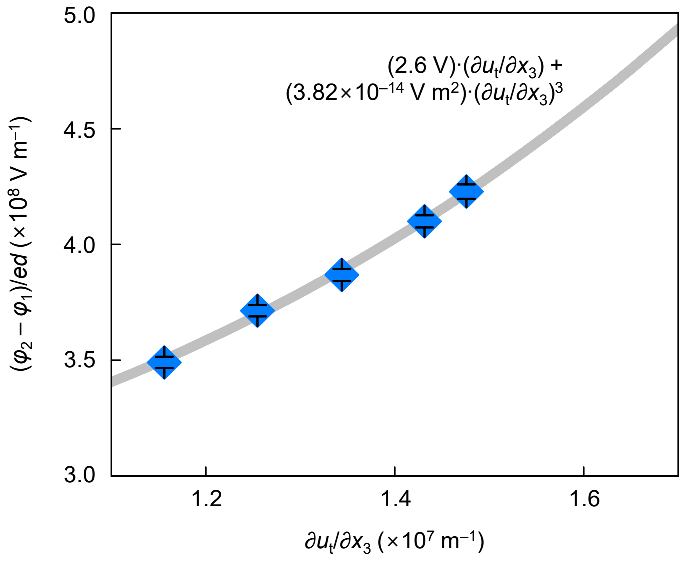

Supplementary Figure 13. Analysis under the assumption of the third-order flexoelectricity. Filled symbols are calculated from Fig. 4a in the main text. The error bars represent the standard deviations of the total electric field, $(\varphi_2 - \varphi_1)/ed$, calculated by fitting the tunnelling spectra in the Supplementary Figs. 2 a-e to the Supplementary Eq. 1. The gray line shows a fit to $f\cdot(\partial u_t/\partial x_3) + g\cdot(\partial u_t/\partial x_3)^3$ with assuming f = 2.6 V.

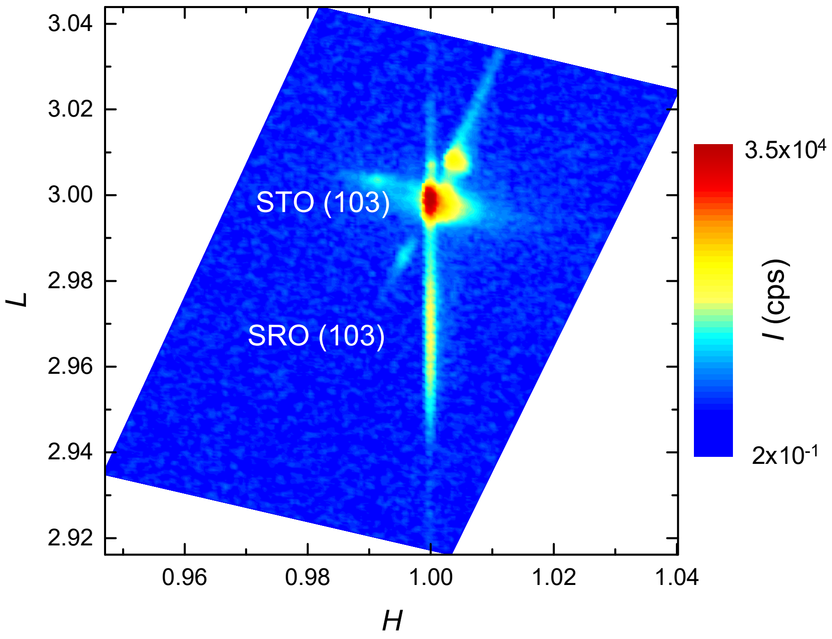

Supplementary Figure 14. Structural characterisation of the nine-unit cell thick $SrTiO_3$ film. Reciprocal space mapping around $SrTiO_3$ (STO) (103) Bragg reflex. Except the peaks from the STO substrate and bottom $SrRuO_3$ (SRO) layer, we did not detect any additional Bragg peak from the STO film. Therefore, this data suggests that our ultrathin STO barrier layer is strain-free. Source data are provided as a Source Data file

Supplementary References

1. Gruverman, A. et al. Tunnelling electroresistance effect in ferroelectric tunnel junctions at the nanoscale. *Nano Lett.* **9**, 3539–3543 (2009).

2. Brinkman, W. F., Dynes, R. C. & Rowell, J. M. Tunnelling conductance of asymmetrical barriers. *J. Appl. Phys.* **41**, 1915–1921 (1970).

3. Khaber, L., Beniaiche, A. & Hachemi, A. Electronic and optical properties of $SrTiO_3$ under pressure effect: Ab initio study. *Solid State Commun.* **189**, 32–37 (2014).

4. Liu, X., Burton, J. D. & Tsymbal, E. Y. Enhanced tunnelling electroresistance in ferroelectric tunnel junctions due to the reversible metallization of the barrier. *Phys. Rev. Lett.* 116, 197602 (2016).

5. Zhuravlev, M. Y., Sabirianov, R. F., Jaswal, S. S. & Tsymbal, E. Y. Giant electroresistance in ferroelectric tunnel junctions. *Phys. Rev. Lett.* **94**, 246802 (2005).

6. Velev, J. P., Burton, J. D., Zhuravlev, M. Y. & Tsymbal, E. Y. Predictive modelling of ferroelectric tunnel junctions. *npj Comput. Mater.* **2**, 16009 (2016).

7. Chu, K. et al. Enhancement of the anisotropic photocurrent in ferroelectric oxides by strain gradients. *Nat. Nanotechnol.* **10**, 972–979 (2015).

8. Naumov, I., Bratkovsky, A. M. & Ranjan, V. Unusual flexoelectric effect in two-dimensional noncentrosymmetric sp^2-bonded crystals. *Phys. Rev. Lett.* **102**, 217601 (2009).

9. Zubko, P., Catalan, G., Buckley, A., Welche, P. R. L. & Scott, J. F. Strain-gradient-induced polarisation in $SrTiO_3$ single crystal. *Phys. Rev. Lett.* **99**, 167601 (2007).
